# Characteristics of Oral Microbiota in Patients with Esophageal Cancer in China

**DOI:** 10.1155/2021/2259093

**Published:** 2021-12-16

**Authors:** Hezi Li, Zhilin Luo, Hong Zhang, Nali Huang, Dong Li, Chengwen Luo, Tianhu Wang

**Affiliations:** Third Affiliated Hospital of Chongqing Medical University, Chongqing, China

## Abstract

Gut microbiota dysbiosis is closely associated with intestinal carcinogenesis, but the oral microbiota of patients with esophageal squamous cell carcinoma who live in high-risk regions in China has not been fully characterized. In the current study, oral microbial diversity was investigated in 33 patients with esophageal squamous cell carcinoma and 35 healthy controls in Chongqing, China, by sequencing 16S rRNA of V3-V4 gene regions. There were statistically significant differences in oral microbiota between esophageal squamous cell carcinoma patients and controls as determined via unweighted pair-group analysis with arithmetic means. At the phylum level, in esophageal squamous cell carcinoma patients, there were comparatively greater amounts of *Firmicutes* (34.0% vs. 31.1%) and *Bacteroidetes* (25.3% vs. 24.9%) and lower amounts of *Proteobacteria* (17.0% vs. 20.1%). At the genus level, esophageal squamous cell carcinoma patients exhibited comparatively greater amounts of *Streptococcus* (17.3% vs. 14.5%) and *Prevotella_7* (8.6% vs. 8.5%) and lower amounts of *Neisseria* (8.1% vs. 10.7%). Using a linear discriminant analysis effect size method, *Planctomycetes* and *Verrucomicrobia* were identified in the esophageal squamous cell carcinoma group. 10 genera were higher abundances identified in the healthy control group, and different 10 genera were identified in the esophageal squamous cell carcinoma group. In the present study, there were significant differences in oral microbial compositions of esophageal squamous cell carcinoma patients and healthy controls. Further longitudinal and mechanistic studies are needed to further characterize relationships between oral microbiota and esophageal squamous cell carcinoma.

## 1. Introduction

Esophageal cancer is one of the most common cancers of the upper gastrointestinal tract, and it is currently the ninth most common cancer overall and the sixth highest cause of cancer deaths globally [1]. Esophageal cancer is more common in males [2].

Esophageal squamous cell carcinoma is more common in Asians, whereas esophageal adenocarcinoma is more common in Europeans [3]. The prevalence of esophageal cancer has increased in recent years but due to a shortage of relevant biomarkers, it is usually not diagnosed early, and there are still few biomarkers to utilize when devising treatment for patients with advanced-stage esophageal cancer. The present study was focused on microbes that may be useful for the tentative diagnosis or early detection of esophageal cancer.

Recent investigations have resulted in the discovery of associations between the oral microbiota and oral diseases, including systemic diseases and cancers [4, 5]. It has been suggested that this is because the oral microbiota plays a key role in aspects of the human immune system, and the immune-inflammatory response of the host to components of the oral microbiome may lead to inflammation [6]. The oral microbiota is almost the same as the esophageal microbiota, and alterations to the oral microbiome may directly affect the esophagus [7]. The upper gastrointestinal tract microbiome and the abundance of bacteria in saliva reportedly only fluctuate slightly at certain time points and then return to their original level during the day [8, 9].

The ongoing development of DNA sequencing technologies and dimensional reduction of algorithm development have made microbiome research cheaper and more controllable. Numerous online microbiome databases have now been established via cross-sectional and longitudinal studies, and they are updated constantly [10]. Notably, however, there are still a lot of unknown factors pertaining to esophageal cancer, particularly with regard to associations with the oral microbiome. The present study was conducted to investigate relationships between the oral microbiome and esophageal cancer in Asians and to generate a corresponding oral microbiome database. Saliva samples were collected, and differences in salivary microbiomes between esophageal cancer patients and controls were investigated via 16S rDNA sequencing.

## 2. Materials and Methods

### 2.1. Study Patients and Specimen Collection

The current case-control study included 33 patients who were diagnosed with esophageal squamous cell carcinoma and underwent upper gastrointestinal examination at the Third Affiliated Hospital of Chongqing Medical University in Chongqing, China, between July 2019 and September 2020. The control group was composed of 35 people who underwent medical examinations at a local hospital. Subjects in the control group had no history of esophagitis or esophagectomy and had not taken antibiotics and proton pump inhibitors in the last month. Patients with rheumatoid arthritis, coronary heart disease, hyperlipidemia, or periodontal conditions were excluded from the study [11]. All the samples were collected during 8:30 a.m. to 11:30 a.m., and the participants were asked to rinse their mouths with flowing water to eliminate food debris and then waited natural salivary secretion. Further details of the exclusion criteria applied are shown in Table 1. Mean and standard deviation are used to describe characteristics of continuous variables except for the age variable. Quartile are used for the age factor. Frequency (percentage) and constituent rations deviation are used to describe characteristics of categorical variable. *T* test or chi-square test are used to compare variables between the two groups. Participants were asked to spit into a sterile tube with cryopreservation with at least 4 ml in 10 min, and they had no oral disease. Saliva samples were collected from all participants and immediately frozen at -80°C using sterile tubes. There were no significant differences in age, gender, or body mass index between the two groups (*p* > 0.05).

### 2.2. Microbial 16S rDNA Sequencing

DNA was extracted from saliva using the FastDNA Spin Kit for Soil (MP Biomedicals, USA) in accordance with manufacturer's instructions, then NanoDrop2000 was used for quality control. Gene amplification was performed with an ABI GeneAmp 9700 instrument. Replicate PCR reactions were performed using modified universal bacterial primers designed to amplify the V3-V4 16S rDNA gene region [12]; 338F (5′-ACTCCTACGGGAGGCAGCAG-3′) and 806R (5′-GGACTACHVGGGTWTCTAAT-3′). The cycling conditions were 95°C for 3 min, followed by twenty-seven cycles of 95°C for 30 s, 55°C for 30 s, and 72°C for 45 s, then a final elongation step of 72°C for 10 min [13]. Every sample were tested in duplicate of three times. PCR products for each sample were detected by 2% agarose gel electrophoresis, then the products were recovered by the AxyPrep DNA Gel Extraction Kit. PCR products were quantified by the Quantus TM Fluorometer. In order to construct library, using PCR to ligation of Illumina barcodes and adaptors. The completed library was sequenced via an Illumina Miseq 2X300bp platform in accordance with Illumina's recommended protocol.

### 2.3. Statistical Analysis

After 16S rDNA sequencing quality control, the length was using a threshold of 50 bp and filtered lower abundance raw reads. Operational taxonomic units (OTUs) were set to measure microbial abundance according to the 16S rDNA conservation sequence. The Silva database (http://www.arb-silva.de) were used to classify the bacteria [14]. A Bayesian algorithm was used to identify the species composition of samples, including microbial phylum, class, order, family, and genus. Prior to diversity comparisons, species accumulation curves were used to assess sample size and species richness.

QIIME software was used to calculate the alpha diversity index, including the observed species index, Chao index, Shannon index, and Simpson index. These indices were used to assess evenness and abundance within groups based on alpha diversity. Rarefaction curve analysis was used to identify species richness and to assess sequence data plausibility. Beta diversity was calculated using weighted and unweighted UniFrac analysis estimated by distances between samples from esophageal squamous cell carcinoma patients and controls. A beta diversity method with principal component analysis, principal coordinates analysis, and unweighted pair-group method with an arithmetic means were used to determine the different oral microbiota in the two groups. Analysis of similarities and Adonis were then used to assess the statistical significance of differences between the esophageal squamous cell carcinoma group and the control group.

The Wilcoxon rank-sum test and phylogenetic investigation of communities were used to identify distinct microbial taxa in the esophageal squamous cell carcinoma group and the control group. Linear discriminant analysis effect size was used to identify taxa that differed in the esophageal squamous cell carcinoma group and the control group, via the nonparametric factorial Kruskal-Wallis sum-rank test function in R (version 3.4.3) [15].

## 3. Results

### 3.1. Characteristics of Subjects and Oral Microbial Composition

A total of 33 patients with esophageal cancer (28 men and 5 women, 56–76 years of age) and 35 age-matched were included in the final analysis after quality control of all samples. All of the subjects in the study were Asian. There was no significant difference in dietary habits between the esophageal squamous cell carcinoma and control groups. Considering the sample size, smoking stratified according to duration, and drinking stratified by alcohol consumption stratification. The smoking ratio was no significant differences between the esophageal squamous cell carcinoma group and control group (*p* = 0.319), the drinking ratio was significant differences between the two groups (*p* < 0.05). With regard to oral condition, the control group reported more toothbrushing and exhibited fewer decayed teeth than the esophageal squamous cell carcinoma group, but these differences were not statistically significant.

**Figure 1:**
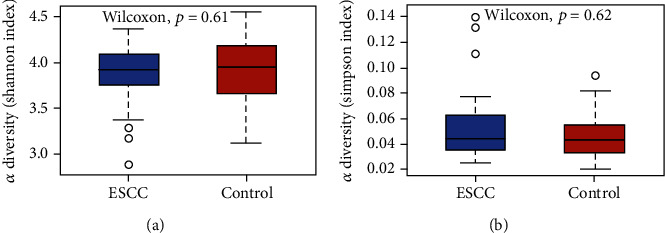
Alpha diversity and richness of oral microbiota in the esophageal squamous cell carcinoma group and the control group determined via the Wilcoxon test. There were no significant differences in alpha diversity between the esophageal squamous cell carcinoma and control groups. (a) Shannon index (*p* = 0.61). (b) Simpson index (*p* = 0.62).

All salivary samples were sequenced to evaluate oral bacterial diversity in the esophageal squamous cell carcinoma group and the control group. There were different 128 OTUs taxa and different 42 OTUs taxa in esophageal squamous cell carcinoma group and control group, respectively. Twenty phyla and 275 genera were identified, and there were no significant differences in alpha diversity between the patient group and the control group. Shannon index was 3.86 and 3.91 in the esophageal squamous cell carcinoma patients and control group, respectively (*p* = 0.61; Figure 1(a)). Simpson index was 0.053 and 0.049 in the esophageal squamous cell carcinoma patients and control group, respectively (*p* = 0.62; Figure 1(b)). The *p* value of observed OTUs was 0.73.

### 3.2. Differences between the Esophageal Cancer Group and the Control Group

**Figure 2:**
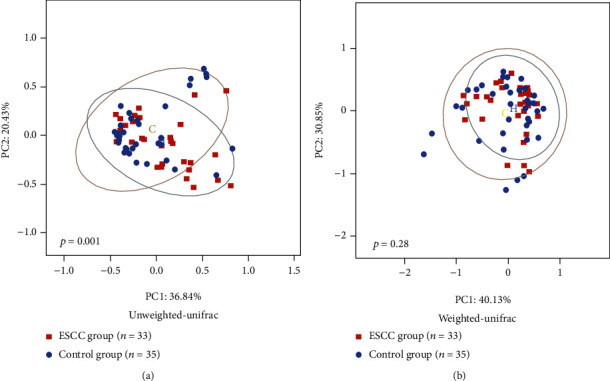
Beta diversity of oral microbiota in the esophageal squamous cell carcinoma group and the control group. There were significant differences in beta diversity between the esophageal squamous cell carcinoma and control groups. (a) Principal coordinate analysis using unweighted-UniFrac of beta diversity. (b) Principal coordinate analysis using weighted-UniFrac of beta diversity.

**Figure 3:**
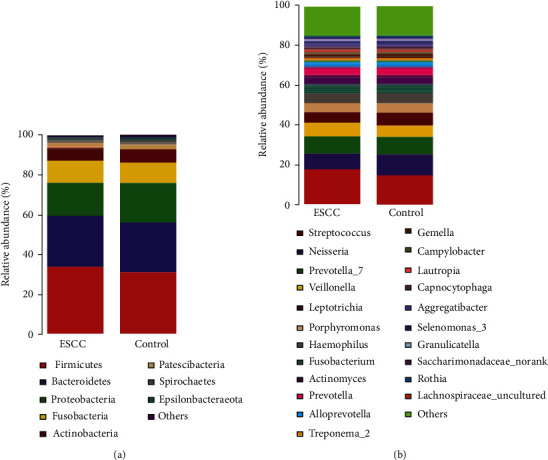
Oral microbial relative abundances at phylum and genus level between the esophageal squamous cell carcinoma and control groups. Identified 8 phylum and 22 genera in two groups, respectively. (a) Phylum level. (b) Genus level.

**Figure 4:**
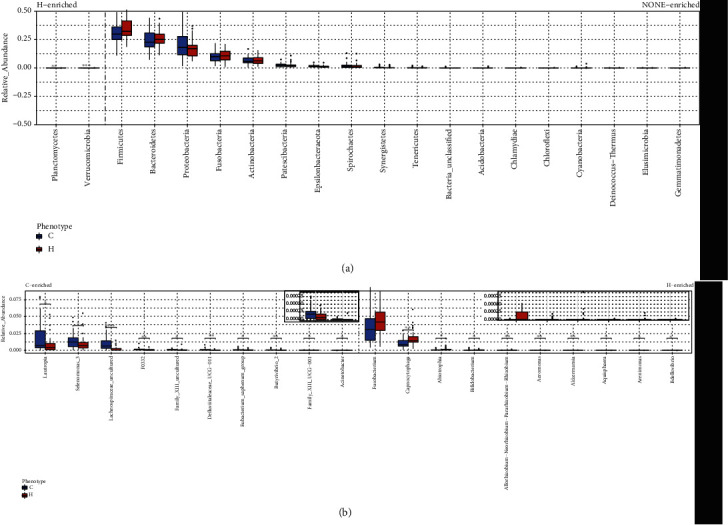
Boxplot representing community differences between the esophageal cancer group and the control group determined via the Wilcoxon test. (a) Phylum level. (b) Genus level. ^∗∗^*p* < 0.01, ^∗∗∗^*p* < 0.001.

In beta diversity analyses, there was a statistically significant difference between the control group and the esophageal squamous cell carcinoma group as determined by the unweighted pair-group method with an arithmetic means. There was no statistically significance difference in clustering between the two groups as determined by the weighted pair-group method with an arithmetic means. The different oral microbiota was identified between esophageal squamous cell carcinoma patients and control groups according to permutational multivariate analysis of variance (Bray-Curtis *p* = 0.031; unweighted *p* = 0.001; weighted *p* = 0.28) and analysis of similarities (Bray-Curtis *p* = 0.048; unweighted *p* = 0.004; weighted *p* = 0.177). These results indicated comparative differences in the abundance of microbiota rather than differences in the types of bacteria in the saliva of the esophageal squamous cell carcinoma group and the control group (Figure 2). At the phylum level, *Firmicutes* (34.0% vs. 31.1%; *p* = 0.17) and *Bacteroidetes* (25.3% vs. 24.9%; *p* = 0.63) were more abundant in the esophageal squamous cell carcinoma group than in the control group, whereas *Proteobacteria* (17.0% vs. 20.1%; *p* = 0.43) were less abundant. At the genus level, *Streptococcus* (17.3% vs. 14.5%; *p* = 0.10) and *Prevotella_7* (8.6% vs. 8.5%; *p* = 0.85) was more abundant in the esophageal squamous cell carcinoma group, but *Neisseria* (8.1% vs. 10.7%; *p* = 0.22) were less abundant (Figure 3). In Mann-Whitney *U* testing to identify differences in bacteria between the two groups, at the phylum level, *Planctomycetes* and *Verrucomicrobia* were identified in the esophageal squamous cell carcinoma group but not in the control group (*p* < 0.05; Figure 4(a)). At the genus level, *Capnocytophaga* were predominant detected in the esophageal squamous cell carcinoma group, and at the family level, *Lachnospiraceae* were predominant detected in the control group (*p* < 0.05; Figure 4(b)).

**Figure 5:**
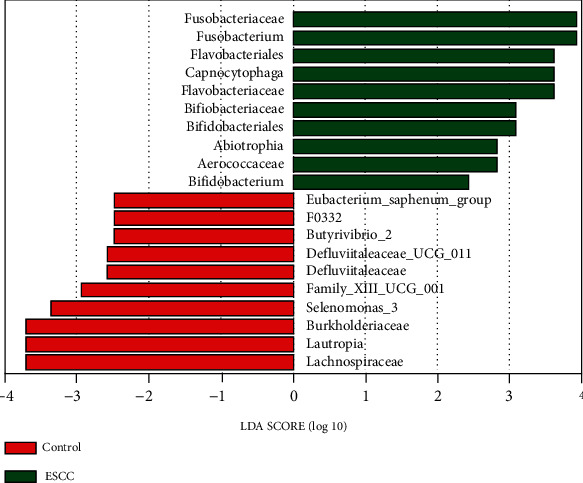
Significant differences in oral microbial taxa between the esophageal squamous cell carcinoma group and the control group. Identified 10 genera in two groups, respectively. Threshold linear discriminant analysis (linear discriminant analysis > 2, *p* < 0.05).

Based on clade abundances at all taxonomic levels, the linear discriminant analysis effect size system for biomarker discovery was used to identify statistically significant biomarkers in the saliva of the two groups. There were 10 genera that were identified in the healthy control group, and different 10 were identified in the esophageal squamous cell carcinoma group (Figure 5). Four phylum-level greater of relative abundance were identified in the esophageal squamous cell carcinoma group, *Bacteroidetes*, *Firmicutes*, *Fusobacteria*, and *Actinobacteria* (*p* < 0.05, linear discriminant analysis > 2). Three phylum-level greater of relative abundance were identified in the control group, *Firmicutes*, *Actinobacteria*, and *Proteobacteria* (*p* < 0.05, linear discriminant analysis > 2). *Capnocytophaga* were significantly more predominant in the esophageal squamous cell carcinoma group than in the control group, and conversely, *Lachnospiraceae* were significantly more predominant in the control group than in the esophageal squamous cell carcinoma group.

## 4. Discussion

Attention to the oral microbiome is increasing in terms of its functions as a predictor and biomarker in human cancers. In other studies investigating the gastrointestinal tract, there have been associations between oral microbiome parameters and digestive tract cancers [16–18]. All subjects in the current study had good dentition and were recorded the number of missing teeth, which has seldom been considered in previous studies investigating the oral microbiota and esophagus microbiome. Short-term dietary intake does not influence salivary microbiome; moreover, the salivary microbiome of individuals does not alter during a day [19, 20]. Some recent studies reported oral microbiota have a linkage to autoimmune diseases, and some associated with cancer, and these findings established a risk prediction model through collected oral microbiome for ESCC [21, 22]. In the current study, there were no statistically significant differences in alpha diversity between the patient group and the control group, but there was a statistically significance difference in relative abundances as determined via beta diversity. Therefore, the oral microbiome may play a key role in the development of esophageal squamous cell carcinoma.

In the present study, OTU phylogeny was compared in saliva samples from the two groups, and the most abundant OTU phyla in the esophageal squamous cell carcinoma and control groups, respectively, were *Firmicutes* (34.0% vs. 31.10%), *Bacteroidetes* (25.3% vs. 24.9%), *Proteobacteria* (17.0% vs. 20.1%), and *Fusobacteria* (10.9% vs. 10.3%). The greater relative abundance of *Firmicutes* and lower relative abundance of *Proteobacteria* in the esophageal cancer group are concordant with Snider et al. [23]. Notably however, Snider et al. [24] reported contrasting results in esophageal adenocarcinoma patients at the phylum level, and their results indicated lower diversity in esophageal adenocarcinoma. They also reported significant differences in microbiomes between patients with Barrett's esophagus and patients with esophageal adenocarcinoma. In Zhao et al. [25], the oral microbiota of 31 esophageal cancer patients in China exhibited more *Firmicutes* and less *Proteobacteria* than controls, which is consistent with the current study. Their findings are that the most significantly increased taxa were six species, while the most significantly decreased taxa were five species. And they concluded that *Prevotella* may be associated with esophageal cancer development.

At the genus level, the most abundant OTUs in saliva samples in the esophageal squamous cell carcinoma group and the control group, respectively, were *Streptococcus* (17.3% vs. 14.5%), *Neisseria* (8.1% vs. 10.7%), and *Prevotella*_*7* (8.6% vs. 8.5%). In the esophageal squamous cell carcinoma group, there was a greater relative abundance of *Streptococcus* and a lower relative abundance of *Neisseria*. In previous studies, *Streptococcus* in the esophagus have been classified into two types, the dominant taxon within the healthy esophagus was *Streptococcus*, and the esophageal adenocarcinoma cascade was reportedly characterized by a shift towards a dominance of Gram-negative bacterial species ([26, 27]). In another study in Chinese Asians, esophageal squamous cell carcinoma patients exhibited less *Streptococcus* at the genus level [28], which differs from the results of the present study at the genus level, but the results were concordant at the phylum level, may be due to the different study designs, sample origins and characteristics of local oral microbiota. Some studies have reported enrichment of specific oral bacterial species such as *Fusobacterium nucleatum* [29], but other studies have reported conflicting results [30]. In one study, protective species were closely connected among different phyla; for example, lower *Neisseria* was associated with higher esophageal squamous cell carcinoma risk [31], which is consistent with the current study. Therefore, the oral microbiome may play a key role in the development of esophageal cancer.

Interestingly, *Planctomycetes* and *Verrucomicrobia* were only in the esophageal squamous cell carcinoma group, and this two phylum microbiomes were core microbiomes. However, the relative of this two phylum microbiomes abundance were low; *Planctomycetes* and *Verrucomicrobia* may be a potential predictor of esophageal squamous cell carcinoma, so there still need some experimental validation. In the present study, *Capnocytophaga* were significantly more prevalent in the esophageal squamous cell carcinoma group, and *Lachnospiraceae* was significantly more prevalent in the control group. *Capnocytophaga* may be a pathogenic bacterium in oral cavity squamous cell cancer [32, 33], and in one case report, *Capnocytophaga* invaded the hyperplasia of an immunocompromised patient [34]. In these studies, a higher abundance of *Capnocytophaga* in the oral cavity was associated with oral diseases, and it may be a promising biomarker for predicting esophageal squamous cell carcinoma. In a previous study, *Lachnospiraceae* functioned as a short-chain fatty acid producer [35], and this family of anaerobic bacteria is reportedly found at relatively low levels in colorectal cancer patients [16, 36]. This result is consistent with the present study, in which there was a higher abundance of *Lachnospiraceae* in healthy individuals. There was a hypothesis indicating that the microbiome is dynamic [37]. Oral microbiome changes in esophageal squamous cell carcinoma patients, whereas oral microbiome remains unchanged in control group. Therefore, the current study indicated that differences in oral microbiota in the current study provide evidence in support of distinctions between esophageal squamous cell carcinoma patients and controls at the genus level and differences in overall ecological structure.

Strengths of the current study are that all patients included were esophageal squamous cell carcinoma; there was no other pathological types of ecological cancer. In order to minimize confounding variables of the microbiome, we designed a rigorous study and followed strict collecting samples standards with health oral cavity. The case-control groups have the same characteristics, such as age, gender, race, and smoking and drinking conditions. These potential variables which may cause confounding bias can be avoided by having the same characteristics between the two groups. Our sample size was relatively large, and clinical studies are still important, and its findings provide hypothesis for following metabolism and microbiome function studies. Limitations of current study are that it describes the different oral microbiome relationship associated with the risk of esophageal squamous cell carcinoma, but further prospective longitudinal studies related to esophageal squamous cell carcinoma development and progression are needed to establish causation. Although models that humanizing immune system are improving, microbial models are still different from human body.

Therefore, multicenter studies are needed in future studies.

## 5. Conclusion

The current study identified direct relationships between esophageal cancer and different oral microbiota, and the functions of the different oral microbiomes were predicted via the construction of a database. The results indicate that differences in the oral microbiota may have causative effects on the risk of esophageal squamous cell carcinoma at phylum level. In addition, the oral microbiota abundance is different between esophageal squamous cell carcinoma and control group at the genus level. Future studies and more mechanistic studies are required in order to control confounding variables.

## Figures and Tables

**Table 1:** General demographic characteristics of the esophageal cancer group and the control group.

Variable	Control	ESCC patients	*t*/*χ*^2^ value	*p*
Age^∗^	61.00 (55.00~70.00)	66.00 (56.00~68.50)	554.00-	0.773
Gender (*n*/%)			3.32	0.069
Female	12 (34.29)	5 (15.15)		
Male	23 (65.71)	28 (84.85)		
BMI (mean ± SD)	23.8 ± 2.0	22.7 ± 2.8	1.84	0.070
Smoking (*n*/%)			0.992	0.319
<35 years	18 (51.43)	13 (37.14)		
≥35 years	17 (48.57)	20 (62.86)		
Drinking (*n*/%)			5.842	<0.05
<100 ml/day	25 (71.43)	14 (42.42)		
≥100 ml/day	10 (28.57)	19 (57.58)		
Frequency of eating spicy food (*n*/%)			4.37	0.358
Never	7 (20.00)	3 (9.09)		
1-3 times/month	6 (17.14)	5 (15.15)		
1-2 times/week	8 (22.86)	4 (12.12)		
3-5 times/week	6 (17.14)	9 (27.27)		
Every day	8 (22.86)	12 (36.36)		
Missing teeth (*n*/%)			6.77	0.148
None	19 (54.29)	12 (36.36)		
1-4	11 (31.43)	12 (36.36)		
5-8	2 (5.71)	2 (6.06)		
9-11	3 (8.57)	2 (6.06)		
≥12	0 (0.00)	5 (15.15)		
Frequency of brushing teeth (*n*/%)			5.17	0.075
Never	0 (0.00)	3 (9.09)		
1 time/day	20 (57.14)	22 (66.67)		
≥2 times/day	15 (42.86)	8 (24.24)		

Note: ^∗^*M* (P25~P75); ESCC: esophageal squamous cell carcinoma; ^∗^*p* < 0.05.

## Data Availability

The raw data in this study are publicly available at https://submit.ncbi.nlm.nih.gov/subs/bioproject/.
